# Metagenomes and metagenome assembled genomes from anaerobic digesters at three Canadian pulp and paper mills

**DOI:** 10.1128/mra.00561-24

**Published:** 2024-10-07

**Authors:** Camilla L. Nesbø, Minqing Ivy Yang, Anupama Achal Sharan, Torsten Meyer, Elizabeth A. Edwards

**Affiliations:** 1 Department of Chemical Engineering and Applied Chemistry and BioZone, University of Toronto, Toronto, Canada; Montana State University, Bozeman, Montana, USA

**Keywords:** pulp mill, metagenome, anaerobic digester, Methanothrix, MAG

## Abstract

We present a dataset of six metagenomes and 323 metagenome assembled genomes (MAGs) describing the microbial community of anaerobic digesters at three Canadian pulp and paper mills. Our objective was to assess the coding potential of the microbial community and obtain draft genomes of key organisms in the digesters.

## ANNOUNCEMENT

In 2017/18, we sequenced the metagenomes of microbial communities in full-scale anaerobic digesters treating wastewater from three Canadian pulp mills. Mill A operates two internal circulation reactors. Mill B has three anaerobic hybrid digesters, each combining a UASB-type reactor and a packed bed reactor. Mill C operates an anaerobic lagoon. Microbial community changes over 1.5 years of monitoring using 16S rRNA amplicon sequencing were previously reported (1). Two DNA samples from each Mill—from summer and winter—were selected for metagenomic sequencing and are reported here. Our objective was to obtain genomic information on organisms in these communities.

**Fig 1:**
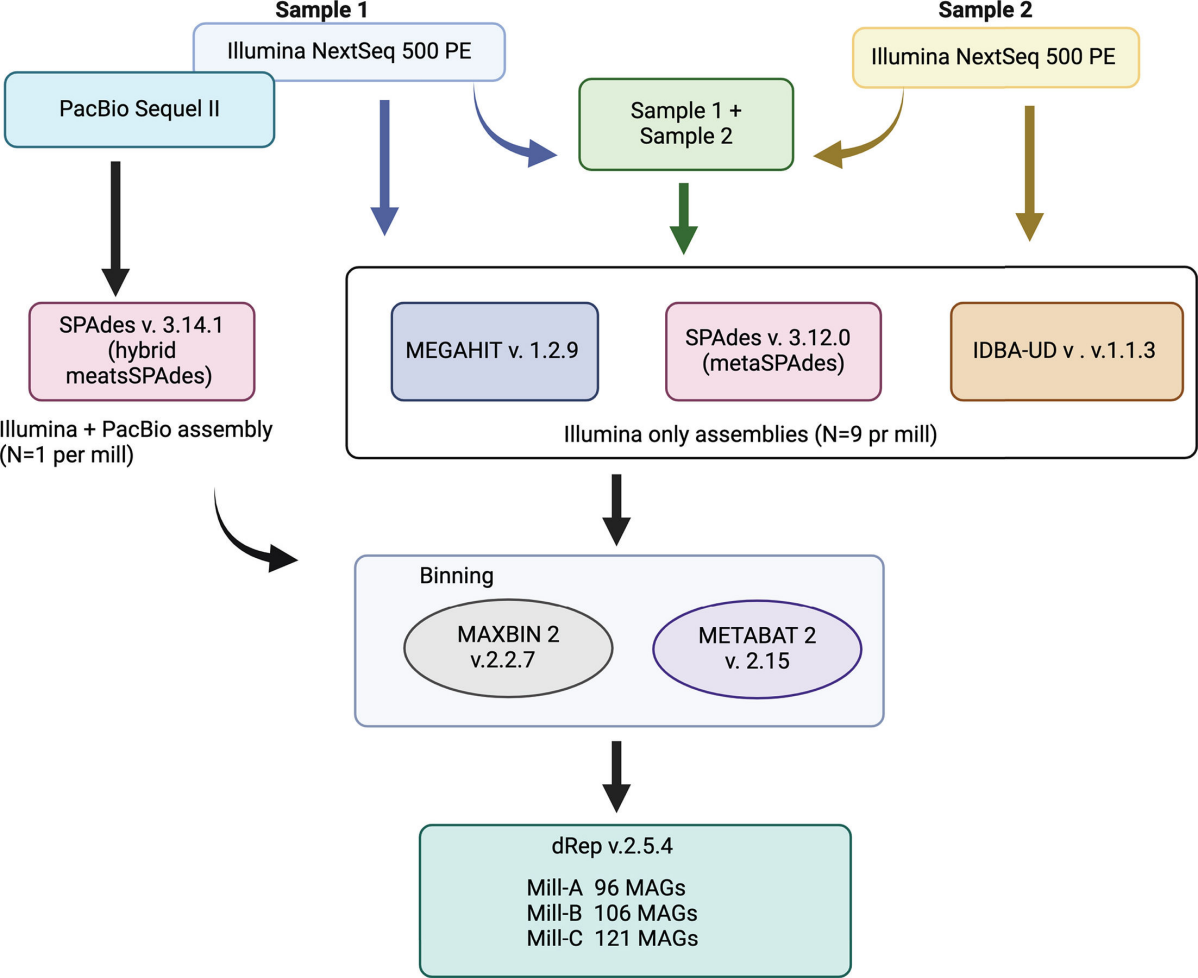
Assembly strategy used in this study. One sample from each mill was sequenced using both Illumina and PacBio (Sample 1; Mill-A = Mill_A_R1_170802_1, Mill-B = Mill_B_AHD1_170726_1, and Mill-C = Mill_C_171204_1), and one sample was sequenced using Illumina only (Sample 2; Mill-A = Mill_A_R1_171227_1, Mill-B = Mill_B_AHD1_180117_1, and Mill-C **=** Mill_C_170825_1). Illumina reads from each sample were assembled individually and in co-assemblies of both Illumina read sets from the same Mill. Three assemblers were used, resulting in nine Illumina-only-assemblies from each mill. In addition, the Illumina and PacBio reads from the same samples were combined in a hybrid assembly. All 10 assemblies from each mill were binned into metagenome assembled genomes (MAGs), and all the MAGs from a mill were compared and dereplicated. The figure was created with BioRender.com.

The samples from reactor 1 at Mill A were collected from a port 6 m above the reactor bottom. For Mill B, the samples were taken from a port 0.5 m above the reactor bottom, and the samples from Mill C were taken from the anaerobic return sludge line (1). After leaving the valve open for enough time to drain the pipes, samples were collected in 0.5-L bottles. The samples were kept cool until DNA isolation. For sample date, see the sample ID in Table 1. Total community DNA was extracted using the Power Soil DNA Isolation Kit (MoBio Laboratories, Carlsbad, CA) from 0.25 g of the sample. For Illumina sequencing, DNA was sheared using Covaris. Libraries were then prepared using the NEB UltraII DNA library prep kit with an insert size of 500 bp prior to sequencing on an Illumina NextSeq sequencer using the NextSeq 500/550 High Output Kit v2.5 (300 Cycles) kit generating paired-end reads of 150 nt at the Centre for the Analysis of Genome Evolution and Function (CAGEF) at the University of Toronto, Canada. DNA from one sample from each mill was prepared using the SMRTbell Express Template Prep Kit 2.0 (Pacific Biosciences, Menlo Park, CA, USA) without size selection and sequenced using PacBio RS at Genome Quebec, Montreal, Canada. Fig. 1 shows the assembly and binning strategy. The Illumina reads were quality-trimmed and assembled using the Anvi’o v6.2 metagenomic snakemake workflow (2). Quality trimming was done using Illumina utilities (3) and read quality control filtering (4) that involves B-tail trimming (low-quality bases at the end of reads), removal of reads containing uncalled bases, and keeping reads where two-thirds of the bases of the first half of the read have quality values of Q ≥ 30. The Illumina reads were then assembled using SPAdes v3.12.0 (metaspades mode (5)), MEGAHIT v1.2.9 (6), and IDBA-UD v1.1.3 (7). PacBio subreads were used in hybrid assemblies with the Illumina reads from the same sample using SPAdes v3.14.1 (hybrid metaspades mode). Assemblies were binned into metagenome assembled genomes (MAGs) using METABAT2 v2.15 (8) and MAXBIN 2 v2.2.7 (9), and the resulting MAGs were compared and dereplicated using dRep v2.5.4 (10) with a pairwise ANI cutoff at 99%, completion >75%, and contamination <25%. The collections of MAGs were combined into one dataset for each mill, and reads were mapped to the MAGs using bowtie2 v2.4.2 to obtain coverage information in Anvi’o v7.1. Taxonomic classification was assigned to each MAG using the GTDB-TK v1.5.1 tool kit (11) and the genome taxonomy database (12). Default settings were used for all software.

**TABLE 1:** Accession numbers and characteristics of metagenomes from anaerobic digesters at three pulp and paper mills^*a*^

Sample ID	SRA and GenBank accession numbers	IMG sequencing project ID	Illumina pairs	PacBio subreads (CCS)	Assembly size / largest contig	N50	No. of contigs
Mill_A_R1_170802_1 (Summer)	SRR24332393 SRR24332392 JASEKO000000000	Gp0458953	23,395,244	8,065,851 (302,997)	610,046,444 / 1,233,384	10,318	151,046
Mill_A_R1_171227_1 (Winter)	SRR24332391 JASEKP000000000	Gp0457370	20,255,390	N/A^*b*^	335,172,164 / 519,347	3,303	183,241
Mill_B_AHD1_170726_1 (Summer)	SRR24332395 SRR24332394 JASEKN000000000	Gp0456687	26,413,714	9,599,482 (293,226)	369,141,812 / 1,990,587	10,353	97,170
Mill_B_AHD1_180117_1 (Winter)	SRR24332396 JASEKM000000000	Gp0457368	26,714,279	N/A	500,831,881 / 506,810	2,597	299,327
Mill_C_170825_1 (Summer)	SRR24332390 JASEKQ000000000	Gp0456574	25,360,675	N/A	503,318,974 / 781,195	2,363	312,146
Mill_C_171204_1 (Winter)	SRR24332389 SRR24332388 JASEKR000000000	Gp0456573	21,751,707	13,126,352 (485,538)	745,876,024 / 1,921,593	8,135	227,352

^*a*^ Assembly statistics are for the SPAdes assemblies of Illumina reads or hybrid assemblies of Illumina and PacBio reads for the samples where PacBio reads were available. The sample date is part of each sample ID; for instance, Mill_A_R1_170802_1 was sampled on 2 August 2017.

^*b*^ N/A indicates not applicable as not all samples were sequenced using PacBio.

Statistics of the SPAdes assemblies are provided in Table 1. Binning of the assemblies resulted in 96 MAGs from Mill-A, 106 MAGs from Mill-B, and 121 MAGs from Mill-C with completeness >75% and contamination =< 25%. Three MAGs correspond to 16S rRNA-*Methanothrix*_feature_319 identified in (1) as a beneficial archaeon that may aid digester recovery after process upsets. These three genomes are very similar across the three mills with the ANI at 99%.

## Data Availability

The SPAdes assemblies, raw Illumina reads, and PacBio reads have been submitted to GenBank under BioProject PRJNA916529. This bioproject also contains the 16S rRNA datasetdata set reported in (1). The assemblies are also available in IMG under Gold study ID Gs0144696. Fasta files for the SPAdes co-assemblies, and all MEGAHIT and IDBA-UD assemblies are available on FigShare (10.6084 /m9.figshare.25555953, 10.6084 /m9.figshare.25555899, 10.6084/m9.figshare.25555968). Fasta files for each of the MAGs are available at FigShare including Excel files with taxonomy and quality statistics for each MAG (https://doi.org/10.6084/m9.figshare.25430992.v3, https://doi.org/10.6084/m9.figshare.25431790.v1, https://doi.org/10.6084/m9.figshare.25429798). The three Methanothrix MAGs have been submitted to GenBank with accession numbers JBCEYP000000000, JBCEYQ000000000, and JBCEYR000000000. A phylogenomic tree of the Methanothrix MAGs can be found on FigShare (10.6084 /m9.figshare.25898647).
